# Relativistic Doppler-boosted ***γ***-rays in High Fields

**DOI:** 10.1038/s41598-018-27122-9

**Published:** 2018-06-14

**Authors:** Remi Capdessus, Martin King, Dario Del Sorbo, Matthew Duff, Christopher P. Ridgers, Paul McKenna

**Affiliations:** 1 SUPA Department of Physics, University of Strathclyde, Glasgow, G4 0NG UK; 2 York Plasma Institute, Department of Physics, University of York, York, YO10 5DQ UK

**Keywords:** Laser-produced plasmas, Plasma-based accelerators

## Abstract

The relativistic Doppler effect is one of the most famous implications of the principles of special relativity and is intrinsic to moving radiation sources, relativistic optics and many astrophysical phenomena. It occurs in the case of a plasma sail accelerated to relativistic velocities by an external driver, such as an ultra-intense laser pulse. Here we show that the relativistic Doppler effect on the high energy synchrotron photon emission (~10 MeV), strongly depends on two intrinsic properties of the plasma (charge state and ion mass) and the transverse extent of the driver. When the moving plasma becomes relativistically transparent to the driver, we show that the *γ*-ray emission is Doppler-boosted and the angular emission decreases; optimal for the highest charge-to-mass ratio ion species (i.e. a hydrogen plasma). This provides new fundamental insight into the generation of *γ*-rays in extreme conditions and informs related experiments using multi-petawatt laser facilities.

## Introduction

The relativistic Doppler effect—the change in the frequency of light due to the relative motion of the source and the observer—is a direct result of special relativity and has a number of consequences for both fundamental and applied sciences. The knowledge of redshifts or blueshifts, occurring whenever a light-emitting source moves away from or toward an observer, has been applied not only to spectroscopic observations of astronomical objects, but also to develop several terrestrial technologies such as Doppler radar^1^. Relativistic Doppler effects have been explored in the laboratory using ultra-intense laser radiation (~10^18^ W.cm^−2^) in order to produce XUV radiation via the relativistic oscillating mirror^2^ and relativistic flying mirror schemes^3^.

At the even higher intensities $(\ge {10}^{23}$ Wcm^−2^)–expected to be achievable using multi-petawatt laser systems such as the Extreme Light Infrastructure (ELI)– it will be possible to produce *γ*-ray sources as well as to enable the exploration of new fundamental processes predicted by classical and quantum electrodynamics (QED)^4–6^. The intensities required for this can be parameterized as ${a}_{{\rm{L}}}\equiv \frac{e{{\rm{E}}}_{{\rm{L}}}}{{m}_{{\rm{e}}}c{\omega }_{{\rm{L}}}}\gg 1$, where *a*_L_ is the normalized field strength, which represents the work (compared to *m*_e_*c*^2^) performed by the field over $\frac{{\lambda }_{{\rm{L}}}}{2\pi }$^7^, where *λ*_L_, −*e*, *m*_*e*_, *ω*_*L*_ and *E*_*L*_ are the laser wavelength, the electron charge, the electron rest mass, the laser frequency and the electric field amplitude, respectively. Such ultra-intense laser radiation will enable the laboratory production of extreme conditions in which collective effects are paramount, accessing physics similar to that encountered in astrophysical events^8–10^. Due to the intense electromagnetic fields involved, quantum electrodynamics effects become important for *χ*_e_ ≳ 0.1. The (Lorentz invariant) QED parameter ${\chi }_{{\rm{e}}}\equiv \frac{{\gamma }_{e}\parallel {{\bf{E}}}_{\perp }+{{\bf{v}}}_{{\rm{e}}}\times {\bf{B}}\parallel }{{E}_{{\rm{S}}}}$ is defined as the electric field in the electron’s rest frame in units of the Sauter-Schwinger field, ${E}_{{\rm{s}}}={m}_{{\rm{e}}}{c}^{3}/e\hslash  \sim {10}^{16}$ Vcm^−1 ^^11^, where *γ*_e_ is the electron Lorentz factor and E_⊥_ is the electric field perpendicular to the electron’s motion. This results in the production of copious amounts of synchrotron-like *γ*-ray emission^12–16^ and electron-positron pairs as *χ*_e_ tends to unity^17–20^. These effects are maximized when the electrons counter-propagate with the laser pulse. They are also enhanced, due to the influence of collective plasma effects, when the plasma is relativistically underdense^15,16,21^; i.e. when the laser frequency becomes higher than the relativistically-corrected-electron plasma frequency ${\omega }_{{\rm{p}},{\rm{e}}}=\sqrt{{n}_{{\rm{e}}}{e}^{2}/{\gamma }_{{\rm{e}}}{m}_{{\rm{e}}}{\varepsilon }_{0}}$, where *n*_e_ is the electron density. Moreover, charged particle dynamics in strong fields are in particularly strongly affected by the radiation reaction (RR) force, that is, how a charged particle interacts with the radiation it emits. In particular, the radiation reaction, which can be interpreted as a friction force in the semi-classical framework (recently shown to accurately describe the average energy loss of the electron population^22,23^), which strongly affects not only the electron dynamics^24–27^ but also those of ions, through the charge-displacement-induced fields^28–30^.

In such ultra-intense laser-plasma interaction regimes, the plasma ions can no longer be considered as background particles, since the quiver electron energy can be comparable with the ion rest mass. It has recently been highlighted that the ion mass affects laser energy absorption and the production of high energy synchrotron-like radiation^16^. In particular, for a given charge state, heavier ions result in a higher yield of *γ*-rays, as well as an enhancement in the radiation emitted in the backwards direction (with respect to laser propagation)^16^. This effect is maximized for a thick plasma layer (i.e. the thickness is such that $l \sim 100{\lambda }_{{\rm{L}}}\gg {l}_{s}$, where *l*_*s*_ = *c*/*ω*_crit._ is the skin length) and relativistically underdense plasma layer. In the case of a thin plasma layer accelerated by a driving force (e.g. the radiation pressure of an ultra-intense laser pulse^31,32^), the ion inertia (due to its mass) could have an impact on the radiation generated by the accelerated electrons through the Doppler effect, resulting from the longitudinal motion of the thin plasma layer. The role of ions in such strongly non-linear regimes remains poorly understood.

**Figure 1:**
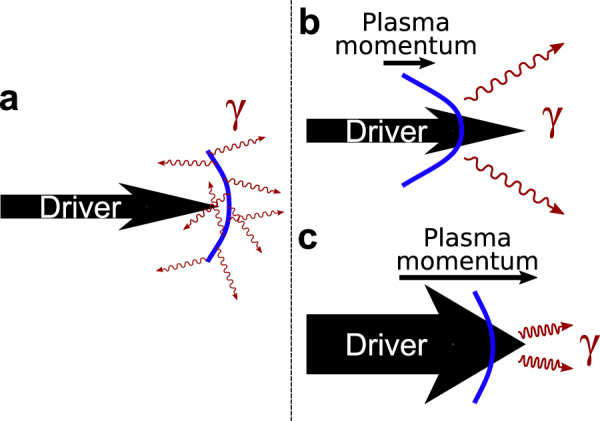
Schematic illustration of a plasma sail pushed by the radiation pressure of a driver. While accelerated by the radiation pressure of a driver (**a**) the electrons of the plasma emit intense synchrotron radiation (from MeV to hundreds of MeV energy) which is Doppler-shifted due to the own motion of the sail. The momentum and dynamics of the sail depend on the transverse extent of the driver and the ion inertia, which has a significant impact on the radiation. When the plasma sail becomes transparent to the driver, the range of angles over which *γ*-rays are produced decreases and the radiation is Doppler-boosted as shown in panels (b and c). Specifically, the larger transverse extent of the driver results in a more Doppler-boosted *γ*-ray emission with an optimal decrease of the angular range of emission for a plasma layer having the highest ion charge-to-mass ratio, i.e. a hydrogen plasma.

In this article, we demonstrate the influence of both the ion charge-to-mass ratio $({\mathscr{Z}})$ and the transverse extent of the driver (*W*_0_) on the high energy synchrotron radiation, emitted by accelerated electrons, as illustrated schematically in Fig. 1. We consider an ultra-intense laser pulse as a driver, striking an overdense plasma layer at normal incidence. The plasma layer is accelerated to relativistic velocities via the laser radiation pressure. In doing so, we show that the relativistic Doppler effect can be tuned through the intrinsic properties of the plasma (charge state and ion mass) and the transverse extent of the driver (see Fig. 1), bringing a new way to control the emission of *γ*-rays.

## Simulation Results

### The laser-piston phase

**Figure 2:**
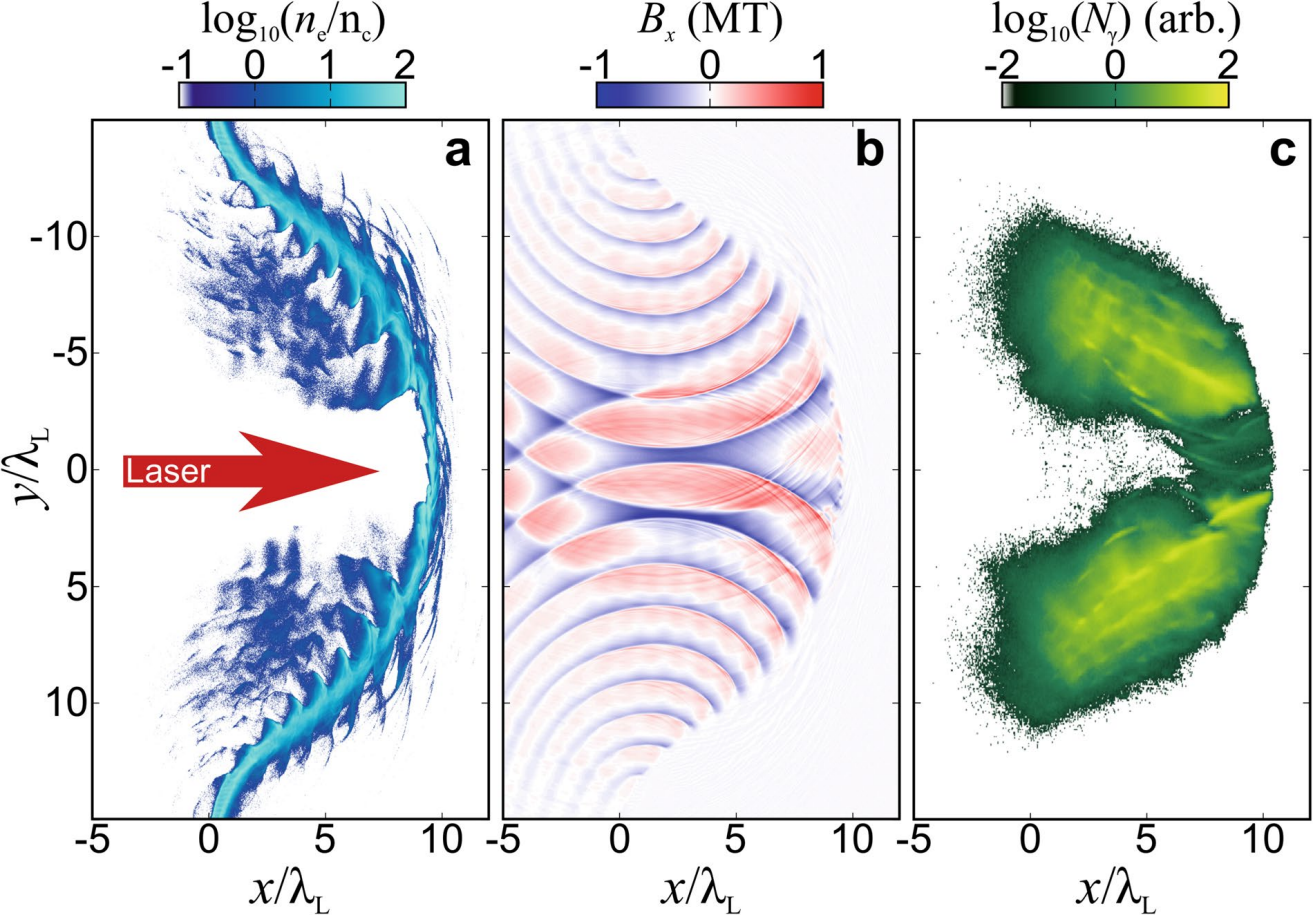
Simulation results of the interaction of an ultra-intense laser pulse with a plasma sail. The laser pulse (*I*_L_ = 10^23^ Wcm^−2^) interacts at *t* = 0 with the plasma slab. The transverse extent of the driver (in this case equal to 15 *μ*m) tends to bend the plasma layer, favoring the generation of an intense longitudinal magnetic field (*B*_*x*_), which guides the radiating electron bunches. (**a**) Electron density normalized to the critical density, *n*_c_. (**b**) Longitudinal magnetic field *B*_*x*_, and (**c**) total number of emitted photons up to *t* = 24 *T*_L_.

Figure 2 shows that the radiating electrons are magnetically-confined into bunches at the edges of the laser pulse, due to the transverse reflection of the surface plasma waves. They have a typical size close to the Larmor radius (*r*_*L*_) such as *r*_*L*_ ~ *cβ*_e_/(*ω*_re_) ~ *γ*_e_*m*_e_*c*/*eB*_*x*_ ≃ 0.8*λ*_L_ (where ${\omega }_{{\rm{re}}}=\parallel {{\bf{p}}}_{{\rm{e}}}\times {{\bf{F}}}_{{\rm{L}},{\rm{e}}}\parallel /{p}_{{\rm{e}}}^{2}$ is the instantaneous electron rotation frequency, *F*_L,e_ is the Lorentz force experienced by the electron, *γ*_e_ ~ *a*_L_ = 200 and *B*_0_ = *m*_e_*ω*_L_/*e* ≃ 1.02 × 10^4^ T is the critical magnetic field) and are separated by one laser period (*λ*_L_) as shown in Fig. 2a,b. In these areas, the longitudinal magnetic field *B*_*x*_ as well as the longitudinal field *E*_*x*_ reaches roughly 40% and 60% of the maximal value of the laser magnetic field (i.e. *B*_*x*_ ≃ 80*B*_0_ ≃ 0.8 MT) respectively and are the dominant fields as the laser field amplitude decreases in the transverse direction due to the Gaussian shape of the laser spot. Therefore *B*_*x*_ induces collective dynamics that influences the synchrotron radiation (also pointed out in a different context in ref.^33^) such that a significant amount of radiated photons are emitted with an energy $\hslash {\omega }_{\gamma }\simeq 0.44{\gamma }_{{\rm{e}}}^{3}\hslash {\omega }_{{\rm{cr}}} \sim 10$ MeV. The generation of the longitudinal magnetic field *B*_*x*_ is due to the reflection of plasma surface waves in the transverse direction (*y*), due to the transverse curvature of the plasma layer due to the finite size of the laser spot. At the centre of the plasma layer, the electrons are expelled from high intensity regions by the ponderomotive force and are accelerated forward, acquiring a strong longitudinal momentum such that *p*_e_ ~ *a*_L_*m*_e_*c*. It has been shown that under these given conditions, the RR force (which can be seen as a friction force, proportional to the instantaneous radiated power; see Methods) can counteract the ponderomotive force and leads to the formation of a confined electron bunch propagating behind the laser pulse front^26^. However, for such an effect to be efficient, the laser intensity has to be beyond ~10^24^ W/cm^2^, which is not the case here. Thus, the ponderomotive force effect dominates the RR force effect, which prevents the formation of a confined electron bunch in the highest intensity area. Thus, most of the synchrotron radiation is produced at the edges of the transverse profile of the pulse and guided by *B*_*x*_ as shown in Fig. 2c.

**Figure 3:**
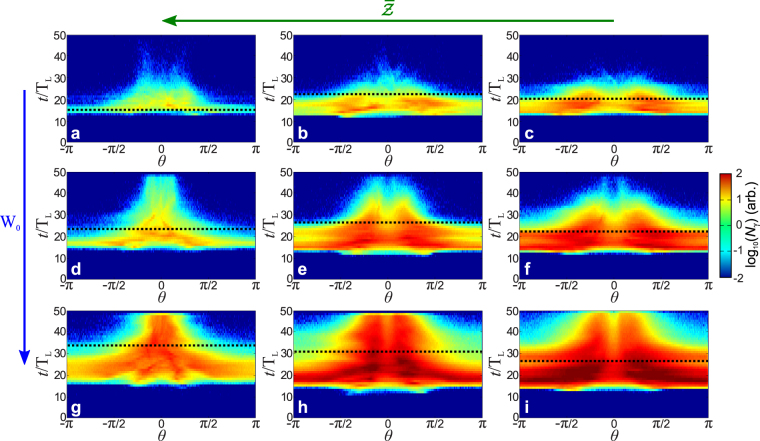
Two-dimensional particle-in-cell simulation results. Angular distribution of 10 MeV photons over time for several ion charge-to-mass ratio $(\bar{{\mathscr{Z}}}\equiv \frac{Z}{{m}_{{\rm{i}}}/{m}_{{\rm{proton}}}})$. (**a–c**) *W*_0_ = 3 μm; (**d–f**) *W*_0_ = 6 μm; (**g–i**) *W*_0_ = 15 μm. (**a**,**d**,**g**), $\bar{{\mathscr{Z}}}=1$; (**b**,**e**,**h**), $\bar{{\mathscr{Z}}}=\frac{1}{2}$; (**c**,**f**,**i)**, $\bar{{\mathscr{Z}}}=\frac{1}{3}$. The black dashed lines corresponds to the time *t*_break_ when the plasma layer becomes relativistically transparent to the laser pulse.

The temporal evolution of the angular distribution of 10 MeV photons shown in Fig. 3, demonstrates clear evidence of the effect of the laser spot size and the ion mass on the synchrotron radiation. As the plasma layer is moving at the average velocity 〈***β***〉, the angular distribution of the emitted radiation is modified through the Lorentz transform $\cos \,\theta =\frac{\cos \,\theta \text{'}+\langle \beta \rangle }{1+\langle \beta \rangle \,\cos \,\theta \text{'}}$^34^. For simplicity, we assume that the transverse motion of the plasma can be neglected compared to its longitudinal motion such that 〈***β***〉 ≈ 〈*β*〉**e**_*x*_ and the related Lorentz factor is written Γ ≡ (1 − 〈*β*〉^2^)^−1/2^. The primes denote variables computed in the instantaneous rest frame of the plasma layer. This special relativity effect enhances the radiation production in the forward direction (*θ* ~ 0^*o*^) and tends to reduce the radiation emitted in the backward direction (|*θ*| ≈ *π*). Since the moving plasma is accelerated this effect is amplified over time as shown in Fig. 3. Moreover, it is optimum for a plasma layer consisting of light ions irradiated by a large spot size as the average velocity of the plasma layer is maximized. As the plasma layer velocity increases over time, the Doppler shift on the radiation becomes more and more significant which can be identified in panels of Fig. 3 through a decrease of the angular emission. Moreover, the synchrotron radiation is Doppler-boosted via the factor ${\mathscr{D}}$ such as

1 $${\mathscr{D}}\equiv {\mathscr{D}}(\langle \beta \rangle ,\theta )=\frac{1}{{\rm{\Gamma }}(1-\langle \beta \rangle \,\cos \,\theta )}$$

It is worth emphasizing why no high energy radiation is emitted before t^*^ ≈ 12 *T*_L_ as shown in Fig. 3. This time can be easily recovered by assuming that the high energy synchrotron radiation generation (i.e. $\hslash {\omega }_{\gamma }\gtrsim {\rm{MeV}}$) becomes important from ${a}_{{\rm{L}}}\approx 100=\frac{{\rm{\max }}[{a}_{L}]}{2}$ (*I* ≃ 10^22^ W/cm^2^) implying ${t}^{\ast }\approx {t}_{{\rm{rise}}}-\frac{{\rm{FWHM}}}{2}\simeq 12{T}_{{\rm{L}}}$ where *t*_rise_ ≃ 18 *T*_L_ is the rise time of the laser field. At the beginning of the interaction, the front of the plasma layer is pushed by the ponderomotive force of the laser pulse which forms an electron density spike in front of the laser that reflects the laser pulse. This forms a double layer structure denoted *laser-piston* and implies the propagation of an electrostatic shock^35^. Due to this structure, the area which includes the shocked plasma without any laser pulse, is separated from the area where there is only the laser pulse and no plasma. The radiation emitted by the electrons is therefore very weak, (i.e. the reflection coefficient in the piston frame of reference is close to unity).

After *t* = t^*^ (which in our case also corresponds numerically to the time for which the *laser-piston* reaches the rear of the plasma layer^35^), a part of the electrons of the plasma layer are piled up at the rear of the target inducing a highly dense electron bunch which reflects the electromagnetic pulse as shown in Fig. 2a. This generates a charge separation field, which accelerates the ions. This regime, known as the *light sail* regime, has been investigated in a number of studies at moderate laser intensities, where the synchrotron radiation emission is non-existent or neglected^31,32^. Over time, the electrons experience higher fields and can escape the electron turning point. They are accelerated both within the rising edge of the laser pulse and due to the ion charge separation layer (Δ_*i*_) ≃ *c*〈*β*〉/*ω*_*p*,*i*_ where ${\omega }_{p,i}=\sqrt{\frac{{n}_{i}{(Ze)}^{2}}{{\gamma }_{i}{m}_{i}{\varepsilon }_{0}}}$ is the ion plasma frequency. Thus, the escaping electrons emit a significant amount of synchrotron radiation as shown in panels of Fig. 3. Whilst the radiating electrons are guided by *B*_*x*_, the charge separation field, *E*_*x*_ boosts the energy of the radiating electrons, rendering their motion more chaotic. Although, the ion mass enhances *E*_*x*_^16,36^, the finite size of the laser spot induces an electric field normal to the plasma layer surface at the edge of the spot which leads to strong electron heating (which increases with ${W}_{0}^{-1}$) and thus an enhancement of the production of synchrotron radiation.

**Figure 4:**
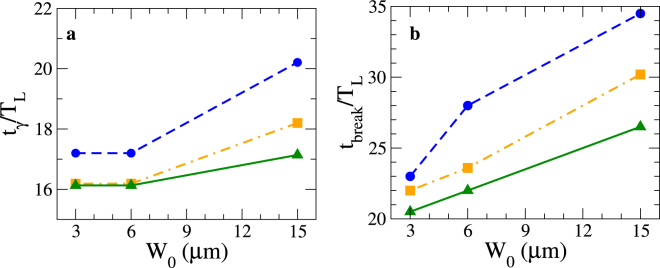
Characteristic times of the simulation results. (**a**) Time corresponding to the peak of the radiated intensity (*t*_*γ*_) and (**b**) the relativistic transparency time (*t*_break_), as a function of the laser spot size (*W*_0_). Dashed blue lines, orange dashed-pointed lines and green lines represent $[\bar{{\mathscr{Z}}}=1]$, $[\bar{{\mathscr{Z}}}=\frac{1}{2}]$ and $[\bar{{\mathscr{Z}}}=\frac{1}{3}]$ plasmas, respectively.

Although it could be supposed that the duration of the synchrotron radiation (*τ*_*γ*_) depends uniquely on the laser pulse duration (*τ*_*L*_), we note that as the laser spot size increases, *τ*_*γ*_ increases significantly as shown in Fig. 3. Indeed, we have ${\tau }_{\gamma ,[{W}_{0}=15{\rm{m}}]}\approx 3\times {\tau }_{\gamma ,[{W}_{0}=3{\rm{m}}]}\simeq 40{T}_{{\rm{L}}}\simeq 140$ fs, higher than *τ*_*L*_ = 30 fs. This can be explained as follows. There are two key processes competing: (i) the transverse expansion of the plasma layer, and (ii) the relativistic longitudinal motion of the plasma. When the transverse expansion of the plasma is important (i.e. involving *small* laser spot sizes, here, less than 6 μm), the electrons are swept away from the high field area before they can interact with the peak of the laser pulse. Thus, for *small* laser spot sizes, the transverse expansion process dominates compared to the relativistic longitudinal motion of the plasma. This implies that the time corresponding to the peak of the radiated intensity (*t*_*γ*_) is less than *t*_rise_, as shown in Fig. 4a. On the contrary, for a $[\bar{{\mathscr{Z}}}=1]$ plasma and a *large* spot size, the relativistic longitudinal motion dominates, which tends to down-shift the laser frequency, thus decreasing the critical density, in the co-moving frame. This implies *t*_*γ*_ ≥ *t*_rise_. We stress that in the case of a negligible transverse expansion the inequality *t*_*γ*_ ≥ *t*_rise_ is always fulfilled, which has been observed in ref.^36^. This emphasizes the influence of the collective plasma effects induced by the laser spot size on the synchrotron radiation. The time interval for which a large amount of radiation is emitted (between 70% and 90%), is strongly enhanced by the size of the spot. This can be identifiable as a *strip* with width roughly equal to *t*_break_ − t^*^, as shown in panels of Fig. 3.

The breakout time *t*_break_ corresponds to the time when the plasma layer becomes relativistically transparent to the laser pulse and is plotted out in Fig. 4b and represented in black dashed lines in Fig. 3. This makes a clear link between the properties of the emitted radiation and the plasma layer evolution. We will see that when the plasma layer becomes relativistically transparent to the electromagnetic pulse, the angular emission of the synchrotron radiation decreases, with a characteristic angle.

**Figure 5:**
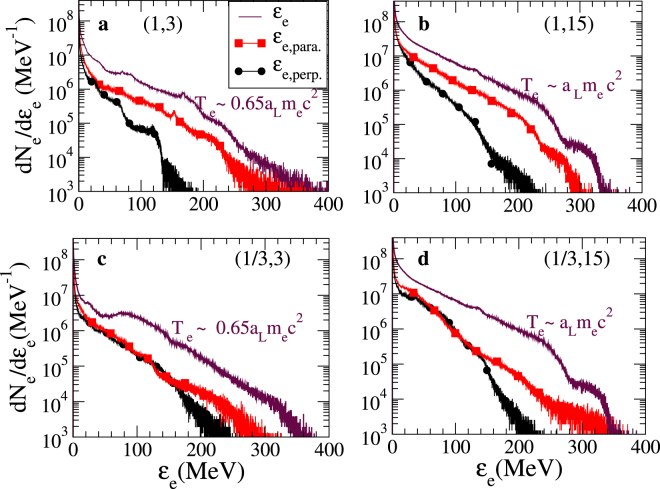
Electron energy spectra. The total energy electron spectra as well as parallel and perpendicular components are plotted out in maroon lines, red squares and black circles, respectively. (**a**) $[\bar{{\mathscr{Z}}}=1]$ plasma and *W*_0_ = 3 μm; (**b**) $[\bar{{\mathscr{Z}}}=1]$ plasma and *W*_0_ = 15 μm; (**c**) $[\bar{{\mathscr{Z}}}=\frac{1}{3}]$ plasma and *W*_0_ = 3 μm; (**d**) $[\bar{{\mathscr{Z}}}=\frac{1}{3}]$ plasma and *W*_0_ = 15 μm. The spectra have been considered at the time corresponding to the maximum synchrotron radiation emission (*t* = *t*_*γ*_), i.e., t = 17 *T*_L_, 20 *T*_L_, 16 *T*_L_ and 17 *T*_L_ respectively. The parallel and perpendicular component of the temperature are defined compared with the direction of the laser wave propagation, i.e., the *x* axis.

It is of interest also to consider how the ion charge-to-mass ratio and the laser spot size affect the electron temperature. Figure 5 shows the electron energy spectra at *t* = *t*_*γ*_ (i.e. time corresponding to the maximum synchrotron radiation emission, plotted out in Fig. 4a), for $[\bar{{\mathscr{Z}}}=1]$ and $[\bar{{\mathscr{Z}}}=\frac{1}{3}]$ plasmas with *W*_0_ = 3 μm and 15 μm. For each case, the full electron energy spectra as well as the perpendicular and the parallel components are plotted out. The hot electron temperature *T*_*e*_ ≈ *a*_L_*m*_e_*c*^2^ = 110 MeV (See Fig. 4) does not significantly depend on the ion charge-to-mass ratio. In the case of *W*_0_ = 3 μm the hot electron temperature is slightly reduced compared to the cases **b** and **d**, where *W*_0_ = 15 μm. However, it is clear that the anisotropy $({\mathscr{A}})$ of the electron distribution function defined as ${\mathscr{A}}\equiv \frac{{T}_{e,\perp }}{{T}_{e,\parallel }}$ depends on these parameters. It tends to increase with ${W}_{0}^{-1}$ and *m*_i_ since the longitudinal motion of the plasma layer is enhanced, which tends to increase the parallel component of the electron temperature. The anisotropy turns out to be minimum for a $[\bar{{\mathscr{Z}}}=1]$ plasma and *W*_0_ = 15 μm, where it is about 0.34 and it is maximum for a $[\bar{{\mathscr{Z}}}=\frac{1}{3}]$ plasma such that ${\mathscr{A}}\approx 1$. Indeed, for a $[\bar{{\mathscr{Z}}}=\frac{1}{3}]$ plasma, the electron distribution function is almost anisotropic for electrons with a kinetic energy (${ {\mathcal E} }_{e}$) less than 150 MeV as shown in Fig. 5c,d. This highlights the important role of both ions and the laser spot size on the features of the electron distribution function and so, on the synchrotron radiation. The development of an analytical model highlighting the role of anisotropicity of the electron distribution on the synchrotron radiation is beyond the scope of this article and is left for future investigations.

### Decrease of the angular emission

As the plasma layer is expanded due to the transverse/longitudinal ponderomotive force, it becomes relativistically transparent to the laser pulse. The threshold of the transparency of the plasma layer may be estimated as^37^

2 $${{\mathscr{D}}}_{{\rm{laser}}}{a}_{{\rm{L}}}(t)=\xi (t)$$

where ${{\mathscr{D}}}_{{\rm{laser}}}\equiv {\mathscr{D}}{(\langle \beta \rangle ,\theta \simeq 0)}^{-1}={(\frac{1-\langle \beta \rangle }{1+\langle \beta \rangle })}^{1/2}$ is the Doppler factor on the laser photons. The shell surface density evolves in time due to the transverse expansion through *ξ*(*t*) = *ξ*_0_/Λ(*t*) where ${\xi }_{0}\equiv \pi \frac{{n}_{{\rm{e}}}}{{n}_{{\rm{c}}}}\frac{l}{{\lambda }_{{\rm{L}}}}$ (see Methods) and Λ(*t*) describes the transverse curvature of the expanded plasma layer and is inversely proportional to the laser spot size as illustrated in Fig. 1. From the relationship (2), we can see that the faster a plasma is moving, the longer it remains opaque to the laser, which is consistent with the values of the characteristic time *t*_break_ shown in Fig. 4b.

**Figure 6:**
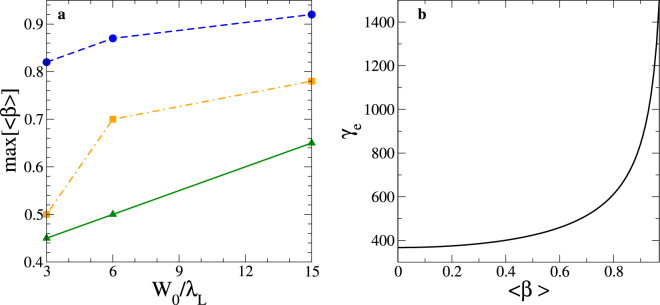
Evolution of the plasma speed and the mean electron Lorentz factor. (**a**) Plasma speed as a function of the laser spot size $(\frac{{W}_{0}}{{\lambda }_{{\rm{L}}}})$ at the transparency time (*t*_break_), computed from simulation results (see methods). Dashed blue lines, $\bar{{\mathscr{Z}}}=1$; orange dashed lines, $\bar{{\mathscr{Z}}}=1/2$; green line, $\bar{{\mathscr{Z}}}=1/3$. (**b**) Mean electron Lorentz factor (4) as a function of the plasma speed during the transparency regime.

For a fixed laser spot size, a $[\bar{{\mathscr{Z}}}=1]$ plasma will be transparent at later times compared to a plasma layer consisting of heavier ions as shown in Fig. 4b. This can be explained as follows. The lighter the ion, the higher the plasma layer velocity. Therefore, due to the Doppler down shift of the laser intensity in the instantaneous rest frame of the plasma layer (i.e. ${I}_{L\text{'}}={{\mathscr{D}}}_{{\rm{laser}}}^{2}{I}_{L}$) the transparency process occurs later on with high $\bar{{\mathscr{Z}}}$. A smaller laser spot size enhances the transverse ponderomotive force, which implies a faster transverse expansion of the plasma, resulting in transparency occurring earlier. This aspect is enhanced with increasing ion mass as the electron heating is maximized. Such statements are consistent with with transparency times plotted in Fig. 4b. In the absence of transverse expansion, and immobile ions, one retrieves the well known threshold of the transparency that is *a*_L_ ≃ *ξ*^31,38^. When the plasma layer becomes transparent to the electromagnetic pulse (i.e. at *t* = *t*_break_), the reflection coefficient drops to zero. This implies not only a negligible radiation pressure but also the breakdown of the longitudinal magnetic field structures, shown in Fig. 2b. The plasma layer is no longer accelerated and we may therefore assume that plasma layer speed attains its maximum value (see Fig. 6a) and becomes constant (i.e. ${\partial }_{t}{\langle \beta \rangle }_{t\ge {t}_{{\rm{break}}}}=0$ and ${\langle \beta \rangle }_{t={t}_{{\rm{break}}}}=\,\max \,[\langle \beta \rangle ]$) and the electromagnetic laser wave propagates now within a relativistically underdense plasma^12,18^. The radiating electrons propagate now in the direction of the laser wave propagation since the longitudinal ponderomotive force dominates. Heuristically, from this stage of the interaction, the laser group velocity (*v*_*g*_) at *t* ⪆ *t*_break_ is very close to the plasma speed, ${\langle \beta \rangle }_{{t}_{{\rm{break}}}}$. In the frame co-moving at 〈*β*〉, the electrons experience a time-varying rotating electric field^18,39^ and have a negligible longitudinal momentum, $({\rm{i}}.\,{\rm{e}}.\,,{p^{\prime} }_{x,e}={\mathscr{O}}({p^{\prime} }_{\perp ,e}))$. Here, *p*_⊥,*e*_ refers to the component of the electron momentum perpendicular to the direction of the laser wave propagation. Assuming a longitudinal motion (*p*_*x*,*e*_  ≫ *p*_⊥,*e*_), from Lorentz transforms, we have *p*_⊥,*e*_ = *p*′_⊥,*e*_ and thus, ${\gamma ^{\prime} }_{e}\approx \frac{{p}_{\perp ,e}}{{m}_{e}c}$. Performing a Lorentz Transform to laboratory frame for the longitudinal component, ${p}_{x,e}=\frac{{p^{\prime} }_{x,e}+{\gamma ^{\prime} }_{e}\langle \beta \rangle {m}_{e}c}{\sqrt{1-{\langle \beta \rangle }^{2}}}\approx \frac{{p}_{\perp ,e}\langle \beta \rangle }{\sqrt{1-{\langle \beta \rangle }^{2}}}$. This results in a decrease of the angular photon distribution, for which the characteristic angle of emission $({\rm{\Theta }}\equiv \arctan (\frac{{p}_{\perp ,e}}{{p}_{x,e}}))$ may be written:

3 $${\rm{\Theta }}=\arctan (\sqrt{\frac{1-{\langle \beta \rangle }_{{t}_{{\rm{break}}}}^{2}}{{\langle \beta \rangle }_{{t}_{{\rm{break}}}}}})\simeq {\langle |{\theta }_{\gamma }|\rangle }_{t\ge {t}_{{\rm{break}}}}.$$

This is consistent with previous work published in ref.^12^. In that paper, an electromagnetic pulse which propagates within a semi-infinite relativistically underdense plasma is considered. The average angle of the radiation emission is expressed through the laser phase velocity such that ${\rm{\Theta }}={({\beta }_{\varphi }^{2}-1)}^{1/2}+{\mathscr{O}}({\beta }_{\varphi }^{2}-1)$. In their study, it is indeed easier to make reference to the phase velocity ${v}_{\varphi }\equiv c{\beta }_{\varphi }\approx {c[1-{(\frac{{\omega }_{p,e}}{{\omega }_{L}})}^{2}]}^{-1/2}$ rather than the plasma velocity itself, which is difficult to estimate in this interaction regime. We stress that from the relationship between the group velocity and the phase velocity, that is *v*_*g*_*v*_*ϕ*_ = *c*^2^ and assuming *v*_*g*_ ≈ 〈*β*〉, one recovers formula (3).

**Figure 7:**
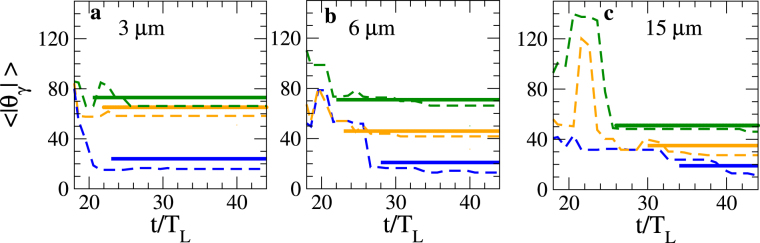
Results of theoretical predictions and numerical simulations. Average angle of the synchrotron radiation 〈|*θ*_*γ*_|〉 as a function of time, for different values of $(\bar{{\mathscr{Z}}},\frac{{W}_{0}}{{\lambda }_{{\rm{L}}}})$. (**a**) *W*_0_ = 3 μm. (**b**) *W*_0_ = 6 μm. (**c**) *W*_0_ = 15 μm. In blue $[\bar{{\mathscr{Z}}}=1]$ plasma. In orange $[\bar{{\mathscr{Z}}}=\frac{1}{2}]$ plasma. In green $[\bar{{\mathscr{Z}}}=\frac{1}{3}]$ plasma. The thick lines and dashed lines correspond to the analytical estimates of Θ (3) defined for *t* ≥ *t*_break_ and the simulation results, respectively.

As the plasma velocity is higher for a $[\bar{{\mathscr{Z}}}=1]$ plasma, it follows that the characteristic angle Θ is smaller for a $[\bar{{\mathscr{Z}}}=1]$ plasma, which is consistent with the values of the mean angle of the synchrotron radiation 〈|*θ*_*γ*_|〉 plotted in Fig. 7. This characteristic angle is numerically defined as $\langle |{{\theta }}_{{\gamma }}|\rangle \equiv \frac{1}{{N}_{y}}\sum _{i=1}^{{N}_{y}}|{{\theta }}_{{\gamma },i}|$, where *N*_*γ*_ is the number of the emitted photons. The analytical values of ${\rm{\Theta }}\simeq {\langle |{\theta }_{\gamma }|\rangle }_{t\ge {t}_{{\rm{break}}}}$ (3) (thick lines) are in very good agreement with the numerical simulation results. The best agreement is obtained in the cases of *W*_0_ = 6 m and 15 μm. In the expression (3) we have assumed that the plasma motion is mainly longitudinal; however, a smaller laser spot size induces a higher transverse ponderomotive force which tends to enhance the transverse speed of the moving plasma.

**Figure 8:**
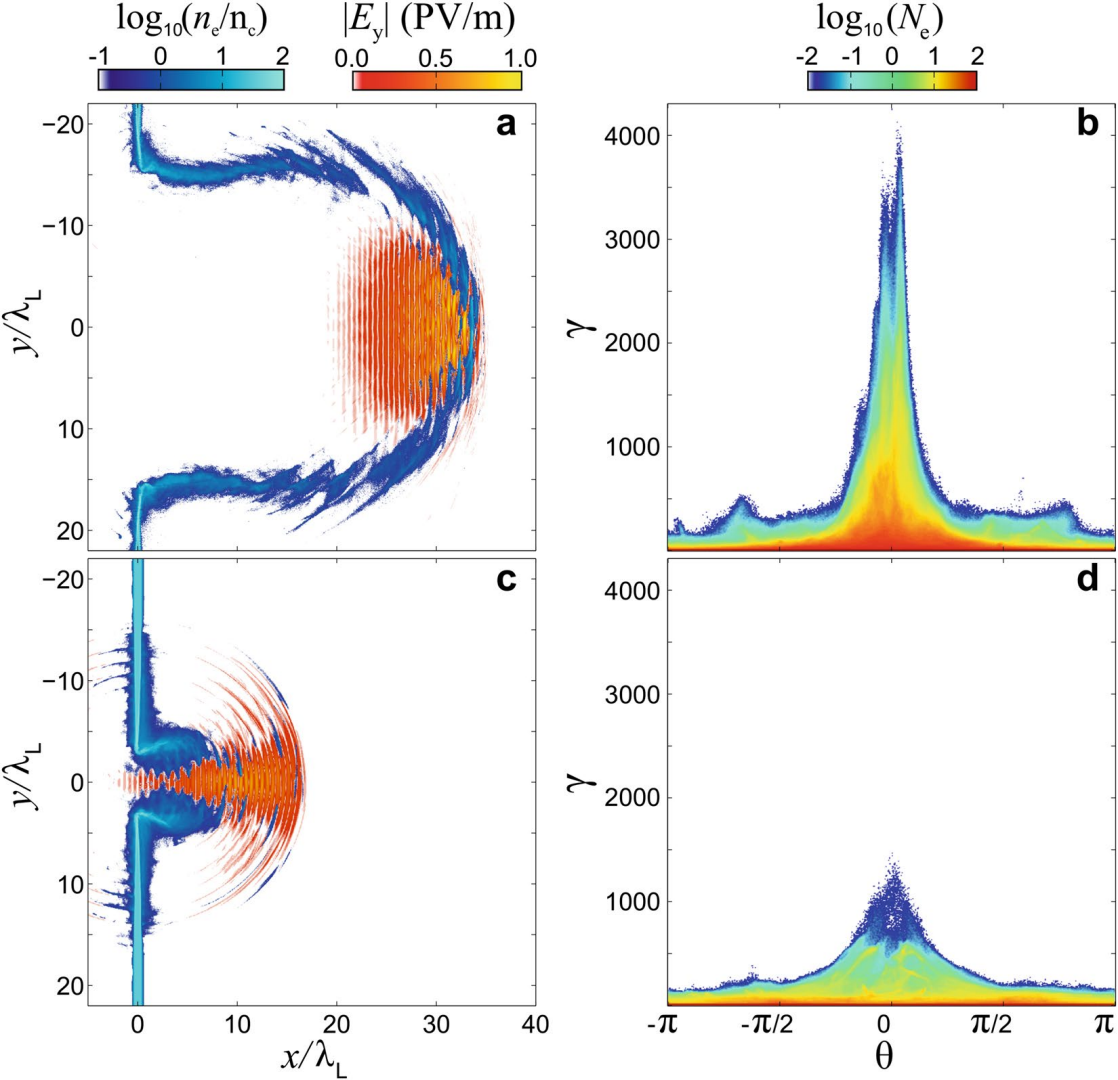
Electron density and laser field behavior during the transparency regime. (**a**) The electron density and laser field. (**b**) The electron energy-angular distribution both for $\frac{{W}_{0}}{{\lambda }_{L}}=15$, $\bar{{\mathscr{Z}}}=1$ at t = *t*_break_ + 13 *T*_*L*_. (**c**,**d**) Same but for $\frac{{W}_{0}}{{\lambda }_{L}}=3$, $\bar{{\mathscr{Z}}}=\frac{1}{3}$.

It is of interest also to examine the electron and laser field behavior during the transparency stage. Figure 8a,c show the electron density and the laser field at *t* = *t*_*γ*_ + 13 *T*_*L*_, for the two *extreme* cases, that is for which (*t*_*γ*_) and ${\langle \beta \rangle }_{{t}_{{\rm{break}}}}$ are maximum and minimum (see Figs 4a and 6a). For a $[\bar{{\mathscr{Z}}}=1]$ plasma with *W*_0_ = 15 μm, the electron population behaves as a dense electron shell moving forward into forward-propagating high-amplitude electromagnetic wave, whereas for a $[\bar{{\mathscr{Z}}}=\frac{1}{3}]$ plasma with *W*_0_ = 3 μm, the electron population is radially expelled by the strong laser field. While the relativistic longitudinal motion of the plasma layer dominates in first case, the transverse expansion process tends to dominate in the second.

The electron energy-angular distributions for both cases are shown in Fig. 8b,d. In the case of an interaction involving both an ultra-strong electromagnetic field and ultra-relativistic electrons such that ${a}_{L}{\gamma }_{e}^{2}\gg {\varepsilon }_{{\rm{r}}{\rm{a}}{\rm{d}}}^{-1}$, the electron Lorentz factor in the co-moving frame may be written ${\gamma ^{\prime} }_{e}\approx {(1+{(\frac{{a}_{L}}{{\varepsilon }_{{\rm{r}}{\rm{a}}{\rm{d}}}})}^{1/2}+\frac{1}{{a}_{L}{\varepsilon }_{{\rm{r}}{\rm{a}}{\rm{d}}}})}^{1/2}$^39^. The parameter *ε*_rad_ = *τ*_*r*_*ω* determines the strength of the radiation reaction force^39^ with ${\tau }_{r}=\frac{{e}^{2}}{6\pi {\varepsilon }_{0}{m}_{e}{c}^{3}}\simeq 6.2\times {10}^{-24}$ s being the radiating time^34^. Performing a Lorentz transform from the co-moving frame to the laboratory frame we obtain an estimate of the mean electron Lorentz factor:

4 $${\gamma }_{e}(t\gtrsim {t}_{{\rm{b}}{\rm{r}}{\rm{e}}{\rm{a}}{\rm{k}}})\approx {[(1+{(\frac{{a}_{L}}{{\varepsilon }_{{\rm{r}}{\rm{a}}{\rm{d}}}})}^{1/2}+\frac{1}{{a}_{L}{\varepsilon }_{{\rm{r}}{\rm{a}}{\rm{d}}}})(\frac{1}{1-{\langle \beta \rangle }_{{t}_{{\rm{b}}{\rm{r}}{\rm{e}}{\rm{a}}{\rm{k}}}}^{2}})]}^{1/2},$$

plotted out in Fig. 6b. This is in good agreement with the electron energy-angular distribution shown in Fig. 8b,d.

**Figure 9:**
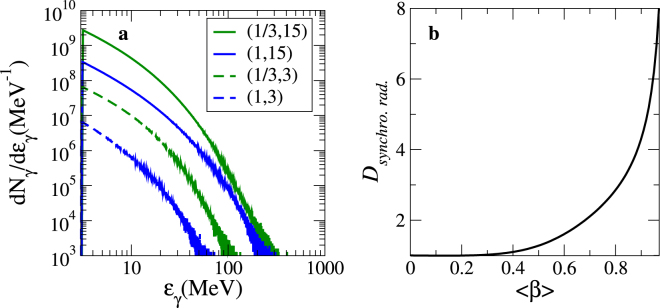
Results of numerical simulations. (**a**) Photon energy spectra above 3 MeV for several values of $(\bar{{\mathscr{Z}}},\frac{{W}_{0}}{{\lambda }_{{\rm{L}}}})$. (**b**) The Doppler-boosted synchrotron factor ${{\mathscr{D}}}_{{\rm{synchro}}.{\rm{rad}}.}\equiv {[{\rm{\Gamma }}(1-\langle \beta \rangle \cos {\rm{\Theta }})]}^{-1}$ (5) once the plasma is transparent to the electromagnetic wave (i.e. *t* ≥ *t*_break_), as a function of the plasma velocity ${\langle \beta \rangle }_{t\ge {t}_{{\rm{break}}}}=\,{\rm{\max }}\,\langle \beta \rangle$.

Figure 9a shows the synchrotron radiation spectra. The influence of both $\bar{{\mathscr{Z}}}$ and *W*_0_ on the synchrotron radiation is significant. Whilst an increase in $\bar{{\mathscr{Z}}}$ enhances the intensity of the radiation (via an increase of the self-consistent fields amplitude)^16^, a larger *W*_0_ enables the emission of photons with energies higher than 100 MeV. From numerical simulation data, we can estimate that the emitted radiation corresponds roughly to a brightness of the order of 10^24^ ph. s^−1^ mm^−2^ mrad^−2^ (0.1% bandwidth)^−1^ (see Methods). Once the plasma is transparent to the electromagnetic wave, the synchrotron radiation is Doppler-boosted by the factor

5 $${{\mathscr{D}}}_{[{\rm{s}}{\rm{y}}{\rm{n}}{\rm{c}}{\rm{h}}{\rm{r}}{\rm{o}}.{\rm{r}}{\rm{a}}{\rm{d}}]}\equiv {\mathscr{D}}(\langle \beta {\rangle }_{{t}_{{\rm{b}}{\rm{r}}{\rm{e}}{\rm{a}}{\rm{k}}}},{\rm{\Theta }})=\frac{1}{{\rm{\Gamma }}(1-\langle \beta {\rangle }_{{t}_{{\rm{b}}{\rm{r}}{\rm{e}}{\rm{a}}{\rm{k}}}}\cos {\rm{\Theta }})},$$

which is plotted out in Fig. 9b. We note further differences between the spectra for which *W*_0_ = 15 μm because the plasma velocity is sufficiently high to induce a significant Doppler-boosted factor (i.e. ${{\mathscr{D}}}_{[{\rm{synchro}}.{\rm{rad}}.,\bar{{\mathscr{Z}}}=1,{W}_{0}=15{\rm{m}}]}\simeq 5$), which makes the highest energy photons generated through a $[\bar{{\mathscr{Z}}}=1]$ plasma and a $[\bar{{\mathscr{Z}}}=\frac{1}{3}]$ plasma comparable.

## Discussion

In the case of a thin plasma layer accelerated by a driving force, we have reported on the influence of the ion charge-to-mass ratio and the transverse extent of the driver on the relativistic Doppler-boosted synchrotron radiation. In the first phase of the interaction, while the radiating electrons are magnetically-confined via the longitudinal magnetic field, the charge separation field enhances the production of radiation. We have demonstrated for the first time that the ion charge-to-mass ratio and the transverse extent of the driver strongly affect the physics of relativistic transparency, which results in both a decrease and an ion-dependent degree of the angular emission of the synchrotron radiation produced. The angular emission is optimal for the highest charge-to-mass ratio ion species, that is, a hydrogen plasma.

The new insight gained into the generation of gamma rays through tuning the relativistic Doppler effect in this important new laser intensity regime paves the way for advancing the development of laser-plasma sources using next generation high power lasers. The experimental evidence of the ion charge-to-mass ratio dependence could be tested at the ELI laser facility with plastic targets such as C-H and C-D or cryogenic hydrogen/deuterium targets. In the collisionless regime obtained with ultra-high intensity and ultra-high energy lasers interacting with low Z targets, the dynamics of the ions is dominated by the ion charge-to-mass ratio. As a first step, C-D plastic target foils can be used to produce a fully ionized C-D plasma with $\bar{{\mathscr{Z}}}$ = 0.5, the same as a deuteron plasma. A fully ionized C-D plasma has $0.5\frac{{Z}_{{\rm{proton}}}}{{m}_{{\rm{proton}}}}$, the same as a deuteron plasma. The angular emission of the *γ*-rays could be evaluated by measuring the angular distribution of the emitted radiation.

As a final remark, our simulation and theoretical results substantially advance our understanding of fundamental ultra-relativistic plasma dynamics and could be relevant for fundamental science and applied sciences. Our results are an essential guide for experimentalists on next generation facilities, allowing them to tune the emission (i.e. duration, intensity, angular emission) of the radiation emitted from the target, by changing the ion charge-to-mass ratio and the laser spot size. Although the generated sources are comparable in brightness (~10^24^ ph. s^−1^ mm^−2^ mrad^−2^ (0.1%bandwidth)^−1^) with large conventional synchrotron sources, they achieve much higher peak energies (10 MeV; i.e. one order of magnitude greater than conventional sources). In particular, they could be used to investigate the emission of the *e*^−^/*e*^+^ pair in the collision of *γ*-ray beams produced with high-intensity lasers^40^. This advances significantly the state-of-the-art of the field providing substantive developments towards ground-breaking papers where these experiments are actually done. Moreover, the use of these plasmas as an intense source of radiation could be useful in order to improve the understanding of phenomena encountered in extreme astrophysical events^41,42^ (i.e. the role of ${\mathscr{Z}}$ and the transverse extent of the driving force: in the dynamics of a radiative collisionless shock^43^, in the Doppler-boosting of the synchrotron emission in *γ*-ray binaries^44^, in the anisotropicity on the gamma-ray burst afterglow emission^45^), which makes a fruitful connection between subfields.

## Methods

### Underpining theory in strong electromagnetic fields

The first covariant formulation was proposed by Dirac in the classical framework, for a charged particle experiencing an intense electromagnetic field, on the basis of energy momentum conservation^46^. However it is well known that the so-called Lorentz-Abraham-Dirac (LAD) equation suffers from anomalies, leading to unphysical solutions. Detailed discussions comparing models are given in references^47,48^. In order to account for the RR force, we consider the Landau Lifshitz equation^34^. Retaining only the dominant term of the RR force proportional to ${\gamma }_{{\rm{e}}}^{2}=1+{p}_{e}^{2}/{m}_{{\rm{e}}}^{2}{c}^{2}$, which can be seen as a friction force, the vectorial form of the Landau Lifshitz equation can be recast as $\frac{d}{dt}{{\bf{p}}}_{e}={{\bf{F}}}_{Le}-\frac{{{\mathscr{P}}}_{\gamma }}{c}{{\boldsymbol{\beta }}}_{{\rm{e}}}$ where, **F**_*Le*_ = −*e*[**E** + *c****β***_e_ × **B**] is the Lorentz force, ${{\bf{F}}}_{r}\equiv -\,\frac{{{\mathscr{P}}}_{\gamma }}{c}{{\boldsymbol{\beta }}}_{{\rm{e}}}=-\,\frac{2{\alpha }_{f}}{3{\bar{\lambda }}_{c}}{\mathscr{G}}({\chi }_{{\rm{e}}}){\chi }_{{\rm{e}}}^{2}{m}_{{\rm{e}}}{c}^{2}{{\boldsymbol{\beta }}}_{{\rm{e}}}$ is the dominant term of the RR force and ${{\mathscr{P}}}_{\gamma }$ is the instantaneous radiated power by one electron. To ensure a semi-classical framework, the dimensionless parameter *χ*_e_ defined as ${\chi }_{{\rm{e}}}=\frac{{\gamma }_{{\rm{e}}}\sqrt{{({\bf{E}}+c{{\boldsymbol{\beta }}}_{{\rm{e}}}\times {\bf{B}})}^{2}-{({\bf{E}}.{{\boldsymbol{\beta }}}_{{\rm{e}}})}^{2}}}{{E}_{{\rm{s}}}}$, which measures the significance of quantum effects, must be less than unity. Here, *E*_s_ = *m*_e_*c*^2^/*e*ƛ_*c*_ is the Schwinger field with ƛ_*c*_ the reduced Compton length. The function ${\mathscr{G}}({\chi }_{{\rm{e}}})\approx {[1+4.8(1+{\chi }_{{\rm{e}}})\mathrm{ln}(1+1.7{\chi }_{{\rm{e}}})+2.44{\chi }_{{\rm{e}}}^{2}]}^{-2/3}$^49^ accounts for the quantum effects which reduces the amplitude of the RR force.

The radiated intensity produced by an electron accelerated in strong electromagnetic fields may be written: $\frac{d{I}_{{e}^{-}}}{d{\boldsymbol{\Omega }}d\omega }={{\mathscr{P}}}_{\gamma }{\boldsymbol{\delta }}({\boldsymbol{\Omega }}-\frac{{{\bf{p}}}_{{\rm{e}}}}{\parallel {{\bf{p}}}_{{\rm{e}}}\parallel })\Upsilon ({\chi }_{{\rm{e}}},{\chi }_{\gamma })$. The parameter ${\chi }_{\gamma }=\frac{e\hslash }{2{m}_{{\rm{e}}}^{3}{c}^{4}}\sqrt{{F}_{b}^{a}{F}_{c}^{b}{k}_{a}{k}^{c}}$ is the associated quantum parameter for emitted photons. The function ϒ(*χ*_e_, *χ*_*γ*_) is defined as $\Upsilon ({\chi }_{{\rm{e}}},{\chi }_{\gamma })$ = $4{(\frac{{\chi }_{\gamma }}{{\chi }_{{\rm{e}}}})}^{2}y{K}_{2/3}(y)+(1-\frac{2{\chi }_{\gamma }}{{\chi }_{{\rm{e}}}})y{\int }_{y}^{{\rm{\infty }}}{K}_{5/3}(x)dx$, where *y* = 4*χ*_*γ*_/[3*χ*_e_(*χ*_e_ − 2*χ*_*γ*_)] and *K*_*n*_ are modified Bessel functions of the second kind. In the semi-classical limit which means *χ*_*γ*_ ≪ *χ*_e_ < 1, the function ϒ(*χ*_e_, *χ*_*γ*_) reduces to the Macdonald function^33^: $\Upsilon ({\chi }_{{\rm{e}}},{\chi }_{\gamma })\to$ ${y}_{c}{\int }_{{y}_{c}}^{{\rm{\infty }}}{K}_{5/3}(x)dx\equiv S(\frac{\omega }{{\omega }_{{\rm{c}}{\rm{r}}}})$ with ${y}_{c}\equiv \frac{\omega }{{\omega }_{{\rm{c}}{\rm{r}}}}={[\frac{3}{2}\frac{{\gamma }_{{\rm{e}}}^{3}\parallel {{\bf{p}}}_{e}\times {{\bf{F}}}_{{\rm{L}},{\rm{e}}}\parallel }{\parallel {{\bf{p}}}_{e}{\parallel }^{2}}]}^{-1}\omega$. For *a*_L_ ≫ 1 the photon formation length becomes small. In this case the radiation is synchrotron-like, with spectrum given by the quantum synchrotron spectrum^49^. It is emitted into a narrow cone within the angle 1/*γ*_e_ with respect to the electron propagation direction and may be modeled by a Dirac function.

### Numerical modeling

To investigate the effects of the laser spot size (i.e. the transverse extent of the driver) and the ion charge-to-mass ratio on the synchrotron radiation, 2D numerical simulations have been performed with the QED-particle-in-cell code EPOCH^50^. Particle-In-Cell (PIC) codes provide a good description of plasma dynamics with reasonable precision and in an acceptable computing time. In particular, PIC codes are useful to study collective effects in plasma which dominate at ultra-high laser pulse intensities. In EPOCH, the interaction of the particles of the plasma (electrons, positrons and ions) with the electromagnetic fields are described by the quasi-classical model of Baier and Katkov^51^, meaning that the particles experience the Lorentz force and photon emission with an emission probability^7^ which induces a recoil, conserving momentum. This recoil gives the quantum equivalent of the RR force^52^.

In order to enable a plasma layer to be accelerated to relativistic velocities while allowing volumetric heating to generate radiation from the accelerated electrons, the initial shell surface density $({\xi }_{0}\equiv \pi \frac{{n}_{{\rm{e}}}}{{n}_{{\rm{c}}}}\frac{l}{{\lambda }_{{\rm{L}}}})$ has to be such that ${\xi }_{0}\lesssim {a}_{{\rm{L}}}$. We thus consider a plasma layer with an initial density equal to 40 *n*_c_ and with thickness *l* = 0.8 μm that is *ξ*_0_ ≃ 100, where ${n}_{{\rm{c}}}={m}_{{\rm{e}}}{\omega }_{{\rm{L}}}^{2}{\varepsilon }_{0}/{e}^{2}$ is the critical density and *λ*_L_ = 1.0 μm. To highlight the role of the ion charge-to-mass ratio and the laser spot size, three plasmas with differing ion charge-to-mass ratios, $\bar{{\mathscr{Z}}}\equiv \frac{Z}{{m}_{{\rm{i}}}\,/\,{m}_{{\rm{proton}}}}$ (1, $\frac{1}{2}$ and $\frac{1}{3}$) and three laser spot sizes, *W*_0_ (3 μm, 6 μm and 15 μm full-width half-maximum), have been considered. To enable the emission of intense and high energy radiation by accelerated electrons whilst enhancing the role of ions, we consider a circularly polarized laser pulse with an intensity peak of 1.1 × 10^23^ W/cm^2^ (i.e. *a*_L_ = *a*_*y*_ = *a*_*z*_ = 200). The spatial and temporal laser profiles are chosen to be Gaussian since this corresponds to the most common experimental profiles. The duration of the laser pulse is 8.6 *T*_L_ (${T}_{{\rm{L}}}=\frac{2\pi }{{\omega }_{{\rm{L}}}}$ = 3.3 fs is the laser period) which corresponds to the typical duration of laser pulses at next generation, multi-petawatt laser facilities, such as ELI. Due to the geometry of the interaction, the QED parameter of electrons *χ*_e_ is small compared to unity and thus the production of *e*^−^/*e*^+^ pairs becomes negligible^7^ and thus, is not considered in this study. It becomes important for laser intensities above 10^24^ W/cm^2 ^^17,18,53^ (i.e. about one order in magnitude higher than in our study). The laser pulse interacts at normal incidence with the plasma layer at *t* = 0 and the simulation box is defined on 200 *λ*_L_ × 50.6 *λ*_L_ using 20000 × 5060 mesh cells.

### Computation of the plasma layer velocity and the transparency time

The mean angle of emission of the high energy synchrotron-like radiation is related to the plasma layer velocity at the transparency time (*t*_*γ*_), when its speed is maximal. Since the analytical computation of the plasma layer velocity is not within the scope of the article, we have obtained this speed through simulation results by measuring the speed of the electron front (see Fig. 2a). To assess the transparency time (i.e., when *n*_*e*_ = *γ*_*e*_*n*_*c*_), both the average electron density and the average electron Lorentz factor have been defined such as $\langle {n}_{e}\rangle$ ≡ $\frac{1}{{W}_{0}}{\int }_{-\frac{{W}_{0}}{2}}^{\frac{{W}_{0}}{2}}{n}_{e}dy$ and $\langle {\gamma }_{e}\rangle$ ≡ $\frac{1}{{W}_{0}}{\int }_{-\frac{{W}_{0}}{2}}^{\frac{{W}_{0}}{2}}{\bar{\gamma }}_{e}dy$ = $\frac{1}{{W}_{0}{n}_{e}}{\int }_{-\frac{{W}_{0}}{2}}^{\frac{{W}_{0}}{2}}$ ${\int }_{{{\mathbb{R}}}^{3}}{f}_{e}{\gamma }_{e}d{{\bf{p}}}_{e}dy$≈ $\frac{1}{{N}_{e}}{\sum }_{k=1}^{{N}_{e}}{\gamma }_{k}(\,-\,\frac{{W}_{0}}{2}\le y\le \frac{{W}_{0}}{2})$, where *f*_*e*_ is the electron distribution function with ${\int }_{{{\mathbb{R}}}^{3}}{f}_{e}d{{\bf{p}}}_{e}\,={n}_{e}$.

### Estimation of the brightness of the ***γ***-ray source

From numerical simulations data, we can estimate the brightness (*B*_*γ*_) as ${B}_{\gamma } \sim \frac{{N}_{\gamma }}{{T}_{{\rm{L}}}}\frac{1}{\sigma }\frac{1}{{\rm{\Omega }}}\frac{1}{0.1 \% \mathrm{BW}}$, where *N*_*γ*_ is the photon number, *σ* the characteristic surface over which the *γ*-ray source is generated and Ω is the solid angle. We assumed that the synchrotron radiation is mainly emitted within the laser spot size (i.e. $\sigma \approx \pi {(\frac{{W}_{0}}{2})}^{2}$) and the solid angle of the source may be written as ${\rm{\Omega }}\approx {\int }_{-{\rm{\Theta }}}^{{\rm{\Theta }}}{\int }_{0}^{\pi }\sin \psi d\psi d\theta =4{\rm{\Theta }}$. *N*_*γ*_ has been estimated by multiplying the number of photons per meter (*N*_*γ*,2*D*_, obtained by the 2D simulation data) by the mesh size (Δx = 10^−8^ m) such that *N*_*γ*_ ≈ *N*_*γ*,2*D*_Δx. Written in conventional units, the brightness writes ${B}_{\gamma }\sim \frac{{N}_{\gamma }}{{T}_{{\rm{L}}}}\frac{1}{\pi {{W}_{0}}^{2}}\frac{1}{{\rm{\Theta }}}\frac{1}{0.1{\rm{ \% }}{\rm{B}}{\rm{W}}}\sim {10}^{24}{\rm{p}}{\rm{h}}.{{\rm{s}}}^{-1}\,{{\rm{m}}{\rm{m}}}^{-2}\,{{\rm{m}}{\rm{r}}{\rm{a}}{\rm{d}}}^{-2}\,{(0.1{\rm{ \% }}{\rm{B}}{\rm{W}})}^{-1}$.
