# Author Correction: Relativistic Doppler-boosted γ-rays in High Fields

**DOI:** 10.1038/s41598-019-43378-1

**Published:** 2019-10-09

**Authors:** Remi Capdessus, Martin King, Dario Del Sorbo, Matthew Duff, Christopher P. Ridgers, Paul McKenna

**Affiliations:** 1 SUPA Department of Physics, University of Strathclyde, Glasgow, G4 0NG UK; 2 York Plasma Institute, Department of Physics, University of York, York, YO10 5DQ UK

Correction to: *Scientific Reports* 10.1038/s41598-018-27122-9, published online 14 June 2018

This Article contains an error in Equation 3.

$$\Theta =\arctan (\sqrt{\frac{1-{\langle \beta \rangle }_{{t}_{{\rm{break}}}}^{2}}{{\langle \beta \rangle }_{{t}_{{\rm{break}}}}}})\simeq {\langle |{\theta }_{\gamma }|\rangle }_{t\ge {t}_{{\rm{break}}}}$$

should read:

$$\Theta =\arctan (\frac{\sqrt{1-{\langle \beta \rangle }_{{t}_{{\rm{break}}}}^{2}}}{{\langle \beta \rangle }_{{t}_{{\rm{break}}}}})\simeq {\langle |{\theta }_{\gamma }|\rangle }_{t\ge {t}_{{\rm{break}}}}$$

